# Correlational analysis of sarcopenia and multimorbidity among older inpatients

**DOI:** 10.1186/s12891-024-07412-2

**Published:** 2024-04-22

**Authors:** Wenjing Xia, Kang Luo, Ziwei Gu, Jianping Hu, Xintong Liu, Qian Xiao

**Affiliations:** Department of Geriatrics, The First Affiliated Hospital of Chongqing Medical University, Chongqing, 400016 China

**Keywords:** Chronic disease, Comorbidity index, Multimorbidity, Sarcopenia

## Abstract

**Background:**

Sarcopenia and multimorbidity are common in older adults, and most of the available clinical studies have focused on the relationship between specialist disorders and sarcopenia, whereas fewer studies have been conducted on the relationship between sarcopenia and multimorbidity. We therefore wished to explore the relationship between the two.

**Methods:**

The study subjects were older patients (aged ≥ 65 years) who were hospitalized at the Department of Geriatrics of the First Affiliated Hospital of Chongqing Medical University between March 2016 and September 2021. Their medical records were collected. Based on the diagnostic criteria of the Asian Sarcopenia Working Group in 2019, the relationship between sarcopenia and multimorbidity was elucidated.

**Results:**

1.A total of 651 older patients aged 65 years and above with 2 or more chronic diseases were investigated in this study, 46.4% were suffering from sarcopenia. 2. Analysis of the relationship between the number of chronic diseases and sarcopenia yielded that the risk of sarcopenia with 4–5 chronic diseases was 1.80 times higher than the risk of 2–3 chronic diseases (OR 1.80, 95%CI 0.29–2.50), and the risk of sarcopenia with ≥ 6 chronic diseases was 5.11 times higher than the risk of 2–3 chronic diseases (OR 5.11, 95% CI 2.97–9.08), which remained statistically significant, after adjusting for relevant factors. 3. The Charlson comorbidity index was associated with skeletal muscle mass index, handgrip strength, and 6–meter walking speed, with scores reaching 5 and above suggesting the possibility of sarcopenia. 4. After adjusting for some covariates among 14 common chronic diseases in older adults, diabetes (OR 3.20, 95% CI 2.01–5.09), cerebrovascular diseases (OR 2.07, 95% CI 1.33–3.22), bone and joint diseases (OR 2.04, 95% CI 1.32–3.14), and malignant tumors (OR 2.65, 95% CI 1.17–6.55) were among those that still a risk factor for the development of sarcopenia.

**Conclusion:**

In the hospitalized older adults, the more chronic diseases they have, the higher the prevalence of sarcopenia. When the CCI is 5, attention needs to be paid to the occurrence of sarcopenia in hospitalized older adults.

**Supplementary Information:**

The online version contains supplementary material available at 10.1186/s12891-024-07412-2.

## Introduction

The concept of sarcopenia was first proposed by Irwin Rosenberg in 1989 [1]. In 2010, the European Working Group on Sarcopenia first published a consensus on sarcopenia and defined it as a geriatric syndrome associated with age–related muscle mass loss, muscle strength loss, and/or physical dysfunction [2]. This age–related disease is known as primary sarcopenia [3]. However, many older adults people have fewer cases of sarcopenia caused by normal aging, and secondary sarcopenia caused by chronic diseases is more common [4]. A meta–analysis showed that the global prevalence of sarcopenia among people ≥ 60 years of age ranged from 10–27% [5]. Another meta–analysis of a community–based older adults population in China estimated the prevalence of sarcopenia to be 17.4% in people ≥ 65 years of age [6]. With the increasing number of older people, sarcopenia has also become one of the major health problems of the older adults.

The incidence of chronic diseases is high in the older adults population and hospitalized older adults people often suffer from various chronic diseases [7]. In 1970, Feinstein, an American scholar, proposed the concept of multimorbidity [8]. Multimorbidity refers to the simultaneous presence of two or more chronic diseases, which can be characterized by the coexistence of multiple somatic diseases, the combination of somatic and mental diseases, the superposition of multiple mental diseases, or the combination of diseases and geriatric syndrome. A cross–sectional study has reported that the prevalence of common diseases in people aged > 60 years is 65.6% [9]. Studies have shown that multimorbidity are strongly associated with age, with prevalence rates of 30% among 45–64 year olds and 65% among 65–84 year olds [10], and that, particularly in the ≥ 80 year old cohort, more than 80% suffer from two or more chronic conditions, and 54% of ≥ 85 year olds suffer from four or more chronic conditions [11]. The prevalence of chronic diseases increases with age in the older adults population [12].

Hospitalized older adults people often suffer from sarcopenia owing to the presence of various chronic diseases [2], and the combination of factors in the multimorbidity state causes problems such as decreased daily activities and malnutrition leading to the development of secondary sarcopenia. Simultaneously, sarcopenia shares many common risk factors with cardiovascular disease, diabetes, respiratory diseases, and chronic kidney disease, thereby increasing the risk of multimorbidity [13]. The interaction between sarcopenia and multimorbidity can lead to debilitating and incapacitating consequences in the older adults, increasing the economic burden of patients, affecting the quality of life in later life, and increasing the mortality rate in the older population. Therefore, this study focuses on the relationship between sarcopenia and multimorbidity in hospitalized older adults to provide a theoretical basis for the early prevention and treatment of sarcopenia in patients with multimorbidity and to avoid the occurrence of a vicious circle.

## Materials and methods

**Fig. 1:**
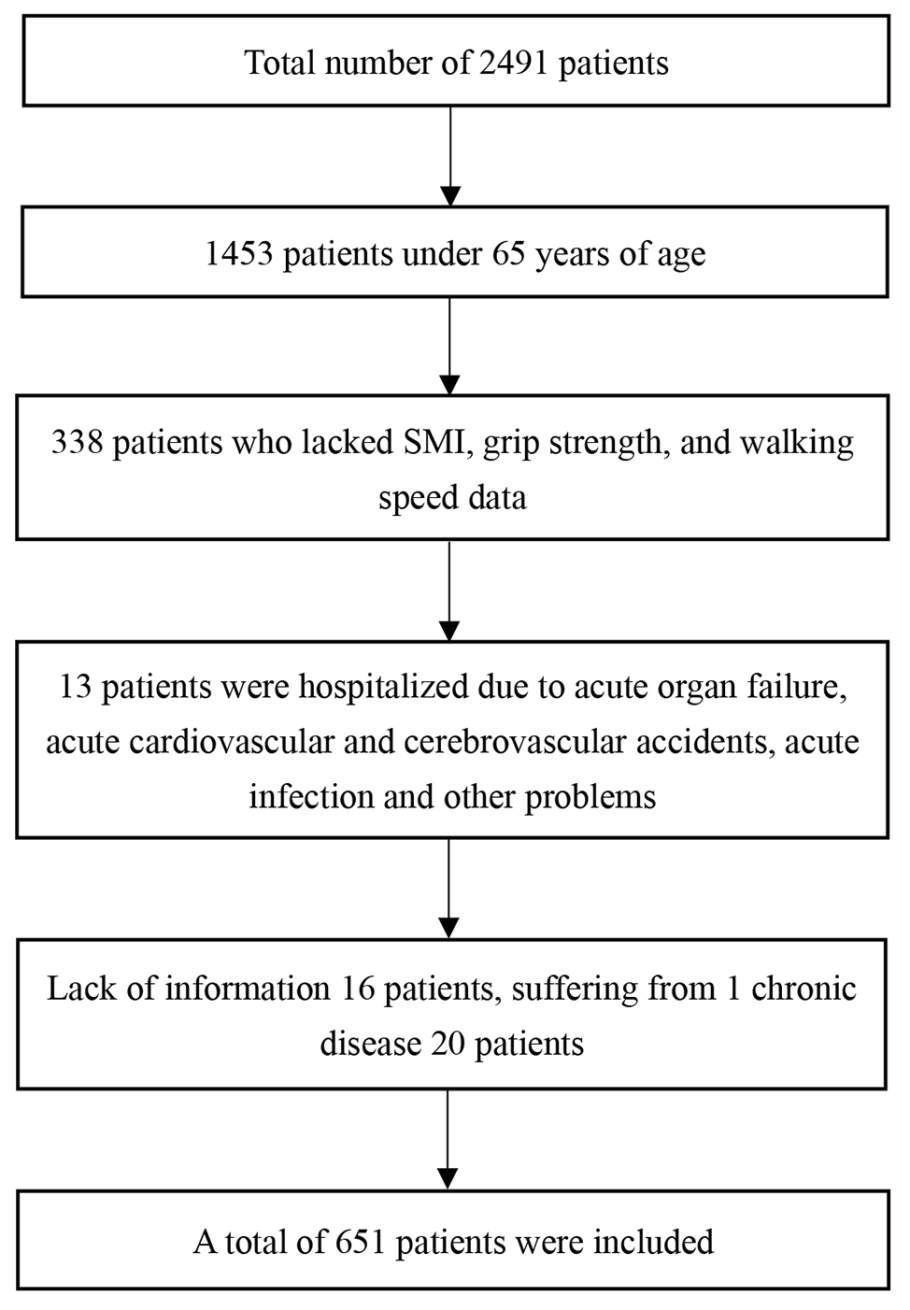
Screening flow chart for inclusion in the population

The subjects were inpatients at the Department of Geriatrics of the First Affiliated Hospital of Chongqing Medical University. The inclusion criteria were as follows: ① age ≥ 65 years old; ② ability to complete the examination required for sarcopenia diagnosis and provide complete data of bioelectrical impedance analysis (BIA), handgrip strength, and 6–meter walking speed; and ③ patients with complete discharge diagnosis. Fourteen categories of chronic diseases were included based on previous studies: hypertension, dyslipidemia, coronary heart disease, diabetes, cerebrovascular diseases (lacunar cerebral infarction, sequelae of cerebrovascular disease, etc.), chronic pulmonary diseases (chronic obstructive pulmonary disease, emphysema, chronic bronchitis, bronchiectasis, etc.), gastrointestinal diseases (peptic ulcers, chronic gastritis, chronic colitis, etc.), chronic liver disease (hepatitis, liver cirrhosis, etc.), chronic kidney disease, metabolic diseases (thyroid dysfunction, Cushing syndrome, etc.), mental disorders (anxiety, depression, mania, schizophrenia, etc.), memory–related diseases (brain atrophy, dementia, Parkinson’s disease, etc.), bone and joint diseases (osteoarthritis, osteoporosis, scapulohumeral periarthritis, etc.), and malignant tumors [14, 15]. The exclusion criteria were as follows: ① patients with acute infectious diseases, acute cardiocerebrovascular diseases, acute organ failure, and so on, and ② those who were unable to complete BIA, handgrip strength test, and 6–meter walk test owing to long–term bedridden condition and sequelae of cerebral infarction. We collected data from 651 hospitalized older patients. The flow chart is as follows (Fig. 1).

### Research methods

Basic information about hospitalization, including sex, age, body mass index (BMI), smoking history, drinking history, education level, and place of residence, was collected. Marital status, including married and other, separated, divorced and widowed are all other. Residence was categorized into large and medium–sized cities and townships and rural areas, and education status was categorized into three levels, First, illiterate and elementary school, second, middle school, secondary school and high school, and third, college and bachelor’s degree and above. Monthly income status was categorized into three levels, low income < 3000, 3000–4999 as middle income, and ≥ 5000 as high income. Ability to perform activities of daily living (ADL) and nutritional risk were assessed according to the nursing staff specialized in geriatrics by means of the Barthel Index (BI) rating scale, and the Mini Nutritional Assessment (MNA) scale. The BI rating scale was classified into four grades, with 100 was classified as no need to be dependent, 61–99 as mildly dependent, 41–60 as moderately dependent, and ≤ 40 as severely dependent. The MNA scale was classified into three grades, >24 as good nutritional status, 17–24 as at risk of malnutrition and <17 as definite malnutrition.

The chronic diseases of each patient were recorded according to the 14 types of common chronic diseases and disease–related Charlson comorbidity index (CCI) [16]. The degree of multimorbidity of patients was evaluated based on CCI. Diseases with 1 point included myocardial infarction, heart failure, peripheral artery obstructive disease, cerebrovascular disease, senile dementia, chronic obstructive pulmonary disease, connective tissue disease, gastroduodenal ulcers, diabetes, and mild liver disease; diseases with 2 points included diabetes if other organs are involved, chronic kidney disease, hemiplegia, leukemia, malignant lymphoma, and tumors; a disease with 3 points was a moderate–to–severe liver disease; and diseases with 6 points were metastatic tumors and AIDS. The scores were adjusted based on age, with 1 point for 50–59 years old and increasing by 1 point for each additional 10 years.

The Asian Working Group on Sarcopenia (AWGS) 2019 has published an expert consensus on the diagnosis and treatment of sarcopenia. The diagnosis of sarcopenia consists of three main areas, muscle mass, muscle strength, and physical performance. The diagnostic criteria for sarcopenia are the presence of low appendicular skeletal muscle mass (ASM) and low muscle strength or physical performance. Those who did not have a decrease in muscle strength or only had a decrease in muscle strength without a decrease in muscle strength and physical performance were non sarcopenia patients. ASM of the extremities was measured and recorded using an Inbody bioelectrical impedance body composition analyzer (Korea). Muscle mass is related to the size of a person’s body, and the larger the size of the person the more muscle mass is usually present, so quantifying the muscle mass requires correcting the absolute value of ASM by the square of the person’s height. Skeletal muscle mass index (SMI) measured by bioelectrical impedance analysis (BIA) was < 7.0 kg/m^2^ for men and < 5.7 kg/m^2^ for women, suggesting a loss of muscle mass. Measurement methods were as follows: Prohibit large amounts of food and water within 2 hours of measurement, prohibit strenuous exercise before measurement, choose a suitable measurement environment, empty urine and feces before measurement, remove shoes and socks, lie flat on the surface of the insulated body, a small amount of alcohol to wipe off the wrist joints, ankles, and other skin dust and dirt, and then take the measurement. The JAMAR grip strength meter (America) for grip strength determination, the test subjects took a standing position, elbows were fully extended, and the use of the grip strength meter in the maximum force Maximum reading of at least 2 trials using the dominant hand in an isometric contraction. Upper extremity grip strength has been widely recognized as an indicator of muscle strength, grip strength < 28 kg for men and < 18 kg for women indicates a decline in muscle strength. 6–meter walking speed to indicate physical performance, step speed measurements, the patient was instructed to walk at a conventional walking speed through a distance of 6 m, without acceleration or deceleration, and measured the time, to assess the patient’s physical performance. The 6–meter walking speed < 1 m/s indicates a decline in physical performance [17].

### Statistical analysis

SPSS 27.0 statistical software was used for data analysis. Descriptive analysis was performed using mean ± standard deviation for measurement data, and the t–test was used for comparing the means between the two groups. The chi–squared test was used for comparing the rates between the two groups for count data. Correlation analysis of the two continuous variables was performed using Spearman correlation analysis. We chose meaningful indicators to be included in the covariates, including age, BMI, ADL, nutritional status, education, and marital status, and model 1 mainly included patients’ own internal influences, while education and marital status added in model 2 were external influences. The influence factors related to sarcopenia were analyzed using Logistic regression analysis. GraphPad Prism 10.0 statistical software was used to do ROC plots for CCI and sarcopenia, as well as logistic regression analysis plots and for chronic disease versus sarcopenia. The difference was considered statistically significant at a *p*–value of < 0.05.

## Results

A total of 651 older adults hospitalized patients were investigated in this study with a mean number of diseases of 3.85 ± 1.34 and a mean CCI score of 5.53 ± 1.86. The cohort consisted of 302 patients with sarcopenia (46.4%) and 349 patients without sarcopenia (53.6%).

### Sarcopenia in the hospitalized older population

Older hospitalized patients with sarcopenia had higher age (82.51 vs. 76.30years), lower BMI (22.35 vs. 25.49 kg/m2), higher number of chronic diseases (4.19 vs. 3.55), higher comorbidity scores (6.49 vs. 4.69), and lower SMI (5.63 vs. 6.72 kg/m2), handgrip strength (16.80 vs. 22.21 kg), and 6–meter walking speed (0.74 vs. 0.99 m/s) were lower. There were statistically significant differences in marital history, ability to perform daily activities, nutritional risk, number of chronic diseases, and CCI score between sarcopenic patients and non–sarcopenic patients, and no statistically significant differences in smoking, alcohol consumption, place of residence, or monthly income (Table 1).

**Table 1:** Comparison of the general information of the patients without and with sarcopenia in this study

	sarcopenia (*n*=302)	Non– sarcopenia (*n*=349)	t/χ^2^	p
Sex [n (%)]			2.32	0.127
male	160(49.4)	164(50.6)		
female	142(43.4)	185(56.6)		
Age (years, $\stackrel{-}{x}$* mean ± SD*)	82.51 ±6.69	76.30 ±6.88	–11.63	0.000
BMI (kg/m^2^, $\overline{x}$*mean ± SD*)	22.35 ±3.51	25.94 ±3.67	11.13	0.000
Smoking history [n (%)]	84(49.1)	87(50.9)	0.70	0.404
Drinking history [n (%)]	54(46.2)	87(50.9)	0.00	0.955
Married [n (%)]	221(43.9)	282(56.1)	5.36	0.021
Place of residence [n (%)]			0.40	0.528
large– and medium–sized cities	268 (46.0)	315(54.0)		
rural and township	34(50.0)	34(50.0)		
Educational level [n (%)]			8.11	0.017
illiteracy and primary school	92(53.5)	80(46.5)		
junior high school and high school	116(40.4)	171(59.6)		
college and bachelor’s degree or above	94(49.0)	98(51.0)		
Monthly income [n (%)]			3.76	0.153
lower income	61(45.2)	74(54.8)		
median income	105(42.3)	143(57.7)		
high income	136(50.7)	132(49.3)		
ADL [n (%)]			91.58	0.000
non–dependent	89(28.6)	222(71.4)		
mildly dependency	148(56.7)	113(43.3)		
moderate dependency	37(82.2)	8(17.8)		
heavy dependence	28(82.4)	6(17.6)		
Nutritional assessment [n (%)]			113.72	0.000
normal nutritional status	123(30.7)	278(69.3)		
risk of malnutrition	138(67.0)	68(33.0)		
malnutrition	41(93.2)	3(6.8)		
SMI (kg/m^2^, $\stackrel{-}{x}$*mean ± SD*)	5.63 ±0.81	6.72 ±0.97	15.43	0.000
Grip strength (kg, $\stackrel{-}{x}$*mean ± SD*)	16.80±6.33	22.21±7.87	9.57	0.000
6–meter walking speed (m/s, $\stackrel{-}{x}$*mean ± SD*)	0.74 ±0.29	0.99 ±0.33	10.08	0.000
Number of chronic diseases ($\stackrel{-}{x}$*mean ± SD*)	4.19 ±1.40	3.55 ±1.21	–6.29	0.000
Stratification of diseases number [n (%)]			36.93	0.000
2–3	103(35.8)	185(64.2)		
4–5	145(50.0)	145(50.0)		
≥6	54(74.0)	19(26.0)		
CCI score ($\stackrel{-}{x}$*mean ± SD*)	6.49 ±1.75	4.69 ±1.52	–14.09	0.000

**Abbreviations**: BMI, body mass index. ADL, activities of daily living. SMI, skeletal muscle mass index. CCI, Charlson comorbidity Index

### Relationship between Sarcopenia and multimorbidity in a hospitalized older population

#### Correlation analysis between Sarcopenia and the number of chronic diseases

The risk of prevalence of sarcopenia with a number of chronic diseases of 4–5 was 1.80 times higher than the risk of prevalence with a number of chronic diseases of 2–3(OR 1.80, 95% CI 0.29–2.50), and the risk of prevalence of sarcopenia with a number of chronic diseases of ≥ 6 was 5.11 times higher than the risk of prevalence with a number of chronic diseases of 2–3 (OR 5.11, 95% CI 2.97–9.08), after adjusting for age, education level, marital status, BMI, ADL, and nutritional status, the risk of prevalence of sarcopenia with a number of chronic diseases of 4–5 was 2.59 times higher than the risk of developing sarcopenia with a number of chronic diseases of 2–3 (OR 2.59, 95% CI 1.65–4.05), and the risk of developing sarcopenia with a number of chronic diseases of ≥ 6 was 5.03 times higher than the risk of developing sarcopenia with a number of chronic diseases of 2–3 (OR 5.03, 95% CI 2.35–10.76) (Table 2).

**Table 2:** Logistic regression analysis of number of diseases with sarcopenia

	Unadjusted			Adjusted		
	OR	95% CI	*p*–value	OR	95% CI	*p*–value
Diseases number						
2–3	1.00			1.00		
4–5	1.80	0.29–2.50	0.001	2.59	1.65–4.05	0.000
≥6	5.11	2.87–9.08	0.000	5.03	2.35–10.76	0.000

Adjusted: adjusted for age, education, marital status, BMI, ADL, nutritional status

The number of chronic diseases is 4–5 and ≥ 6, respectively, which is 2.05 times (OR 2.05, 95% CI 1.45–2.90) and 4.18 times (OR 4.18, 95% CI 2.16–8.10) lower than the population with 2–3. After adjusting for factors such as age, education level, marital status, BMI, ADL, and nutritional status, the population with 4–5 and ≥ 6 chronic diseases had 2.52 times (OR 2.52, 95% CI 1.71–3.71) and 3.86 times (OR 3.86, 95% CI 1.85–8.04) lower handgrip strength compared to the population with 2–3, respectively.

The SMI of the population with ≥ 6 chronic diseases is 1.27 times lower than that of 2–3 (OR 1.27, 95% CI 0.91–1.76). After adjusting for age, education level, marital status, BMI, ADL, and nutritional status, the SMI of the population with ≥ 6 chronic diseases are 3.47 times lower than that of 2–3 (OR 3.47, 95% CI 1.90–6.32). The SMI of the population with 4–5 chronic diseases is also lower than that of 2–3, but there is no statistical difference.

The population with ≥ 6 chronic diseases had a 2.03–fold lower 6–meter walking speed compared to 2–3 individuals (OR 2.03, 95% CI 1.15–3.60). After adjusting for age, education level, marital status, BMI, ADL, and nutritional status, the population with ≥ 6 chronic diseases had a lower 6–meter walking speed compared to 2–3 individuals, but there was no statistically significant difference. The number of chronic diseases is 4–5, which is lower than 2–3 with a 6–meter walking speed, but there is no statistical difference. (Table 3)

**Table 3:** Logistic regression analysis of number of diseases with sarcopenia components

	Unadjusted			Adjusted		
	OR	95% CI	*p*–value	OR	95% CI	*p*–value
Diseases number						
low muscle mass						
2–3	1.00			1.00		
4–5	1.27	0.91–1.76	0.158	1.54	1.00–2.36	0.050
≥6	3.47	1.90–6.32	0.000	3.16	1.46–6.83	0.003
low muscle strength						
2–3	1.00			1.00		
4–5	2.05	1.45–2.90	0.000	2.52	1.71–3.71	0.000
≥6	4.18	2.16–8.10	0.000	3.86	1.85–8.04	0.000
low physical performance						
2–3	1.00			1.00		
4–5	1.07	0.77–1.49	0.684	1.06	0.73–1.53	0.780
≥6	2.03	1.15–3.60	0.015	1.69	0.88–3.23	0.115

Adjusted: adjusted for age, education, marital status, BMI, ADL, nutritional status

#### Correlation analysis between Sarcopenia and Charlson comorbidity index

According to Spearman correlation analysis, there is a weak correlation between SMI and CCI (*r*=–0.14, *p* = 0.000), and CCI is also correlated with grip strength (*r*=–0.22, *p* = 0.000) and 6–meter walking speed (*r*=–0.33, *p* = 0.000) (Table 4). The additional scatterplot demonstrates this even more [see Additional file 1–3].

**Table 4:** Linear correlation analysis between the components of sarcopenia and Charlson comorbidity index

	r	*p*–value
CCI		
SMI	–0.14	0.000
Grip strength	–0.22	0.000
6–meter walking speed	–0.33	0.000

**Fig. 2:**
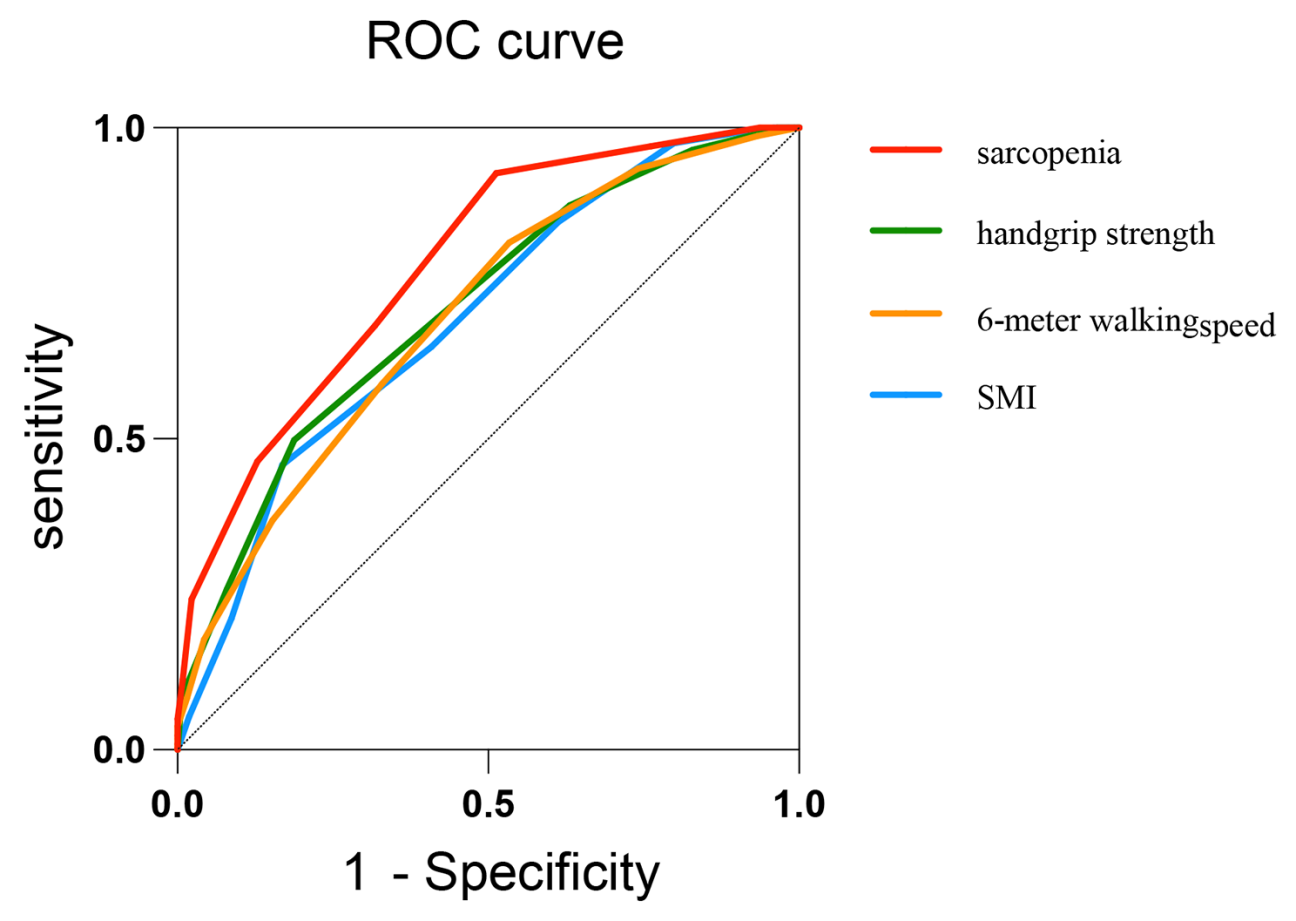
ROC curve of sarcopenia and diagnostic parameters with Charlson comorbidity index

We plotted the ROC curves of SMI, handgrip strength, walking speed, and sarcopenia with CCI (Fig. 2). If CCI is used to evaluate low muscle mass, the area under the curve (AUC) is 0.691 (*p* = 0.000), and the corresponding critical value is 5 when the Youden index is maximum. If diagnosed with low muscle strength, the AUC is 0.708 (*p* = 0.000), and the corresponding critical value is 5 when the Youden index is maximum. If diagnosed with low physical function, the AUC is 0.695 (*p* = 0.000), and the corresponding critical value is also 5 when the Youden index is maximum. When we use CCI to evaluate the presence or absence of sarcopenia, the AUC is 0.777 (*p* = 0.000), and the corresponding critical value is 5 when the Youden index is maximum. The additional table shows the problem more clearly [see Additional file 4].

**Fig. 3:**
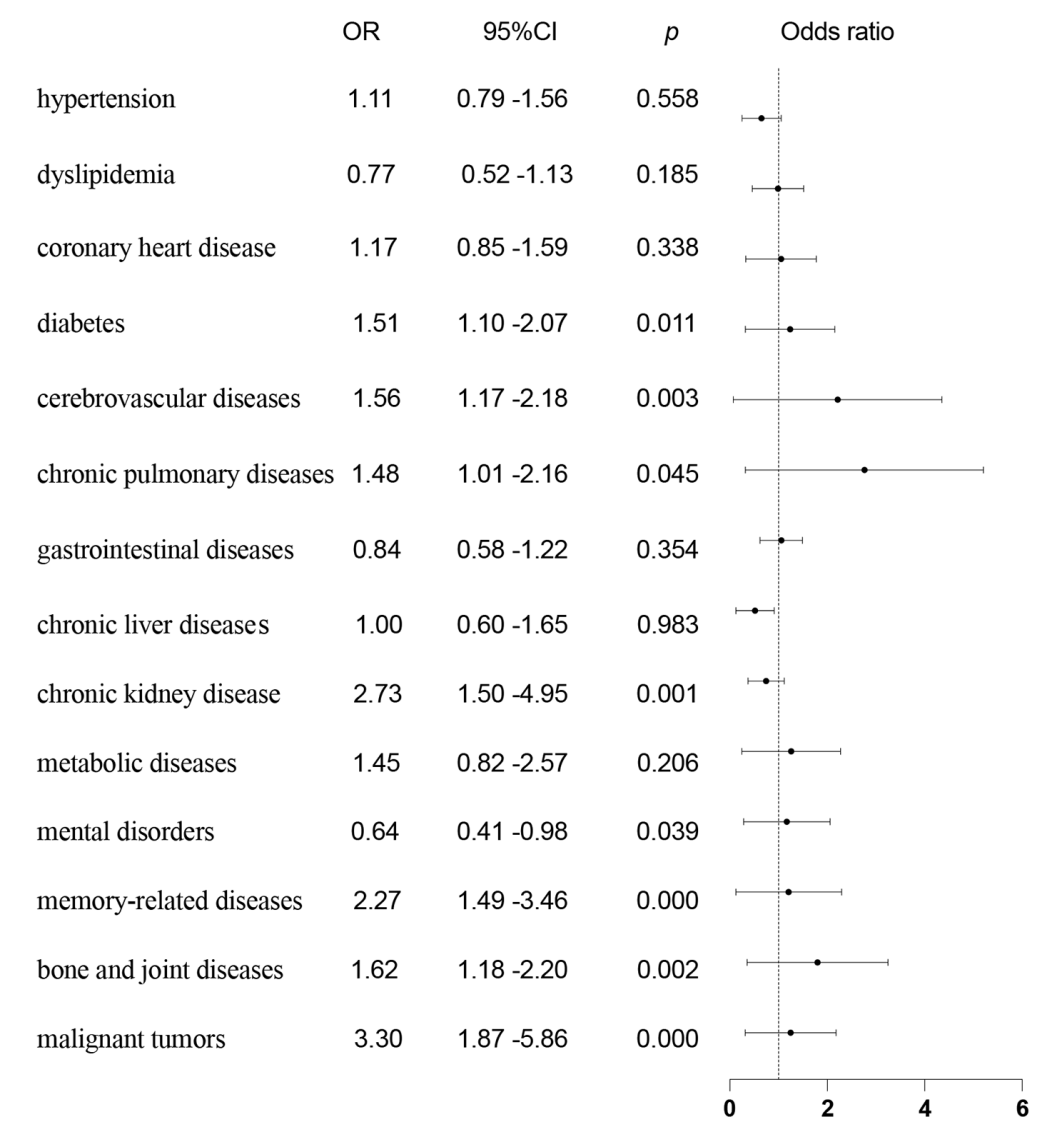
Univariate regression analysis of the combinations of chronic diseases and sarcopenia

Hypertension, coronary heart disease, diabetes, dyslipidemia, cerebrovascular diseases, chronic pulmonary diseases, gastrointestinal diseases, chronic liver disease, chronic kidney disease, metabolic diseases, mental disorders, memory–related diseases, bone and joint diseases, and malignant tumors (yes = 1, no = 0) were identified as independent variables. Logistic regression analysis was performed with sarcopenia as the dependent variable (yes = 1, no = 0). Univariate logistic regression showed that the combination of diabetes (OR 1.51, 95% CI 1.10–2.06), cerebrovascular disease (OR 1.60, 95% CI 1.17–20.18), chronic lung disease (OR 1.48, 95% CI 1.01–2.16), chronic kidney disease (OR 2.73, 95% CI 1.50–4.95), memory–related diseases (OR 2.27, 95% CI 1.49–3.46), bone and joint diseases (OR 1.62, 95% CI 1.18–2.20), and malignant tumors (OR 3.30, 95% CI 1.87–5.86) were risk factors for sarcopenia (Fig. 3), while mental diseases were risk factors for sarcopenia protective factors (OR 0.64, 95% CI 0.41–0.98). Single factor logistic regression significant indicators were selected as independent variables, and sarcopenia as dependent variable. Multivariate logistic regression analysis was conducted, including age, BMI, ADL, and nutritional status as covariates, showing that diabetes (OR 3.25, 95% CI 2.06–5.16), cerebrovascular disease (OR 2.06, 95% CI 1.36–3.19), bone and joint disease (OR 1.99, 95% CI 1.30–3.06), malignant tumor (OR 2.56, 95% CI 1.13–5.80) is a risk factor for sarcopenia. After being included in marital status and education, diabetes (OR 3.20, 95% CI 2.01–5.09), cerebrovascular disease (OR 2.07, 95% CI 1.33–3.22), bone and joint disease (OR 2.04, 95% CI 1.32–3.14), and malignant tumor (OR 2.65, 95% CI 1.17–6.55) are still risk factors for sarcopenia (Table 5).

**Table 5:** Multivariate regression analysis of the combinations of chronic diseases and sarcopenia

		Model 1			Model 2	
	OR	OR95% CI	*p*–value	OR	OR95% CI	*p*–value
diabetes	3.25	2.06–5.16	0.000	3.20	2.01–5.09	0.000
cerebrovascular diseases	2.06	1.36–3.19	0.001	2.07	1.33–3.22	0.001
bone and joint diseases	1.99	1.30–3.06	0.002	2.04	1.32–3.14	0.001
malignant tumors	2.56	1.13–5.80	0.024	2.65	1.17–6.55	0.020

Model 1: adjusted for age, BMI, ADL, nutritional status

Model 2: adjusted for age, education, marital status, BMI, ADL, nutritional status

## Discussion

Estimating the cumulative number of chronic diseases is a common method for evaluating multimorbidity [7]. The morbidity of sarcopenia with 2–3, 4–5, and ≥ 6 chronic diseases was 35.8%, 50.0% 和 74.0%, respectively. The risk of sarcopenia in hospitalized patients with ≥ 6 chronic diseases and 4–5 chronic diseases is 5.1 times and 1.8 times higher than that of 2–3 chronic diseases, respectively. Therefore, it can be concluded that the more chronic diseases older adults hospitalized patients have, the higher the incidence of sarcopenia may be. Some studies also suggest that multimorbidity may increase the risk of sarcopenia. A cross–sectional study by Smith et al. on 14,585 older adults people aged ≥ 65 years in low and middle–income countries found that ≥ 2 diseases were associated with 1.49 times (95% CI 1.02–2.19) and 2.52 times (95% CI 1.53–4.15) higher odds of sarcopenia and severe sarcopenia, respectively, compared with no chronic disease [18]. Veronese et al. conducted a 12–year cohort study using data from the longitudinal study on aging in the UK (ELSA) and found that among 2873 older adults individuals aged ≥ 60, the incidence of sarcopenia in comorbid patients at baseline was approximately twice that of no multimorbidity (OR 2.06, 95% CI 1.61–2.62). After adjusting for confounding factors, the results were also statistically significant (OR 1.23, 95% CI 1.01–1.61) [19]. Different from the above studies, the population in this study is older inpatients with heavy disease burden. After screening, the number of patients with only one chronic disease in the sample size is very small. Because the lack of data will affect the consistency and accuracy of statistical analysis, it is impossible to effectively compare the difference between non-comorbidities and comorbidities, so we only included older inpatients with comorbidities. In addition, for the diagnosis of sarcopenia, the above studies used an anthropometric equation related to height, weight, age and gender (ASM=0.193×weight+0.107×height−4.157×sex −0.037× age −2.631) to indirectly estimate muscle mass. In this study, bioelectrical impedance analysis was used to evaluate muscle mass, more accurate than equation estimation.

We also found that the number of chronic diseases was 2.0 and 4.1 times lower for those with 4–5 and ≥ 6 compared to those with 2–3, respectively. Dong et al. using data from the China Health and Retirement Longitudinal Study (CHARLS), showed that among 28,368 middle–aged and older adults aged ≥ 40 years, the prevalence of multimorbidity was higher among those with low handgrip strength compared to those with high handgrip strength [20]. A cross–sectional study by Yorke et al. of 5,877 people ≥ 50 years of age from the 2008 Health and Retirement Study (HRS) data in the United States showed that handgrip strength declined progressively with the number of chronic conditions, and was more pronounced in the presence of ≥ 3 chronic conditions [21]. All of the above studies examined the relationship between multimorbidity and handgrip strength, and similar to the findings of the present study, patients with multimorbidity had decreased handgrip strength and may have decreased more severely with increasing number of chronic diseases. The difference is that the present study is more comprehensive in applying both muscle mass and physical performance to the diagnosis of sarcopenia, and we also found that an increased number of chronic diseases was associated with low muscle mass and low physical performance, whereas the present study discusses the relationship between muscle mass and physical performance and the number of chronic diseases in relatively few studies, which may be due to the fact that in the older age group, the decline in muscle strength is more pronounced than that of muscle mass, and that physical performance is easily is influenced by multiple factors and is also not often used in isolation to assess sarcopenia.

CCI, which quantifies complications based on the number and severity of diseases, is the most commonly used multimorbidity assessment method [16]. We concluded that there is a correlation between CCI and SMI, handgrip strength, and walking speed, and that when the CCI reaches a score of 5 and above, there may be sarcopenia. In a study of 168 older adults ≥ 65 years of age, Gong et al. found that higher comorbidity scores were associated with a greater risk of sarcopenia, and they diagnosed sarcopenia using the thoracic skeletal muscle index (TSMI), which is derived from the total muscle area at the level of the twelfth thoracic vertebrae divided by the square of the height, and found that there was a significant linear negative correlation between the TSMI and CCI, and that there was also a significant negative correlation between TSMI and CCI and a significant negative correlation between step speed and CCI. They also found that when the CCI exceeded 6.5 points, there was a need to be concerned about a decrease in skeletal muscle mass [22]. Differences from the above studies differ in that we found that in the hospitalized older population, a CCI of more than 5 points requires attention not only to the decline in skeletal muscle mass, but also at the same time to the presence or absence of a decline in muscle strength and physical performance, and to be alert to the development of sarcopenia. Our study utilized the latest diagnostic criteria of AWGS 2019, which not only assessed muscle mass and physical performance, but also included data on muscle strength, making the diagnosis of sarcopenia more comprehensive, and more studies are still needed in the future to explore the relationship between CCI and sarcopenia, and to discover the appropriate threshold of comorbidity index to guide and apply in the clinic, so as to provide a strong theoretical support for the early prevention of sarcopenia in patients with multimorbidity. Theoretical support for the early prevention of sarcopenia in patients with multimorbidity.

Furthermore, in addition, our study observed that among the common chronic diseases in the older adults, diabetes mellitus, cerebrovascular disease, bone and joint diseases, and malignant tumors were independent risk factors for the development of sarcopenia after adjusting for relevant influencing factors. Similar to the results of this study, Dodd et al. also showed that musculoskeletal/trauma disorders (OR 2.17, 95% CI 2.11–2.23), endocrine/diabetes mellitus (OR 1.49, 95% CI 1.45–1.55), and neurologic/psychiatric (OR 1.39, 95% CI 1.34–1.43) were independently associated with probable sarcopenia among people 40–70 years old association [23]. Insulin resistance, the cause of type 2 diabetes mellitus, inhibits multiple signaling pathways in the body, degrading skeletal muscle proteins and accelerating the loss of skeletal muscle mass [24]. Bones and muscles have strong interactions and their structure and function decline with age [25]. Loss of muscle strength and mass during aging leads to structural changes in the bone microstructure and decreases mineral density, resulting in bone loss [26]. Osteoarthritis patients may suffer from joint pain and stiffness, resulting in limited daily activities and inevitably leading to muscle atrophy and loss of function [27]. Cerebrovascular disease can lead to adverse consequences such as prolonged bed rest or somatic movement disorders, causing the release of multiple chronic inflammatory factors in the body, reduced physical activity leading to muscle wasting. Patients with malignant tumors can exacerbate muscle damage due to the high catabolic state in the body, inflammation, decreased physical fitness, anorexia, and factors related to anticancer therapy, leading to sarcopenia [28]. A study based on the CHARLS has reported that hypertension (OR 1.22, 95% CI 1.09–1.38), chronic lung disease (OR 1.23, 95% CI 1.07–1.42), heart diseases (OR 1.15, 95% CI 1.00–1.33), mental disorders (OR 2.21, 95% CI 1.23–3.96), and arthritis (OR 1.21, 95%CI 1.07–1.35) are associated with the possible development of sarcopenia in middle–aged and older people aged ≥ 45 years [29]. However, the difference lies in the fact that our study was conducted on an inpatient older population and included a wider range of chronic diseases, which were more severe and complex.

Our study has some limitations. Our study population was only the hospitalized older population, and we did not discuss the community older population and the nursing home population, the results are susceptible to sampling error, and we need to further expand the sample size and the sample to cover the population before conducting the study. Simultaneously, we included 14 chronic diseases that are common in older patients, but there are still some diseases that are not involved, and CCI still needs further research and refinement as far as the older population is concerned. Some common degenerative diseases in the older adults, such as Parkinson’s, hypertension, and other common diseases, were not included in the assessment, and more comprehensive multimorbidity assessment tools need to be explored in the future. Finally, this is a cross–sectional study, and the inference of causality between variables needs to be confirmed in additional cohort studies.

## Conclusions

The greater the number of chronic diseases in hospitalized older patients, the greater is the prevalence of sarcopenia. Individualized prevention and treatment programs should be developed early depending on the individual needs of the multimorbidity patient so as to avoid the occurrence and development of sarcopenia, especially with diabetes, cerebrovascular diseases, bone and joint diseases, and malignant tumors.

### Electronic supplementary material

Below is the link to the electronic supplementary material.

Supplementary Material 1

Supplementary Material 2

Supplementary Material 3

Supplementary Material 4

## Data Availability

The datasets used during the current study are available from the corresponding author on reasonable request.

## Abbreviations

ADL: Activities of daily living
ASM: Appendicular skeletal muscle mass
AUC: Area under the curve
AWGS: Asian Working Group on Sarcopenia
BI: Barthel index
BIA: Bioelectrical impedance analysis
BMI: Body mass index
CCI: Charlson Comorbidity Index
CHARLS: China Health and Retirement Longitudinal Study
HRS: Health and Retirement Study
MAN: Mini nutritional assessment
SMI: Skeletal muscle mass index
TSMI: Thoracic skeletal muscle index
