# Supplementary material for: Correlational analysis of sarcopenia and multimorbidity among older inpatients
Source: BMC Musculoskelet Disord. 2024 Apr 22;25:309. doi: 10.1186/s12891-024-07412-2 (PMC11034126; doi:10.1186/s12891-024-07412-2)

Additional file 3 Scatterplot of the correlation between 6-meter walking speed and Charlson comorbidity index


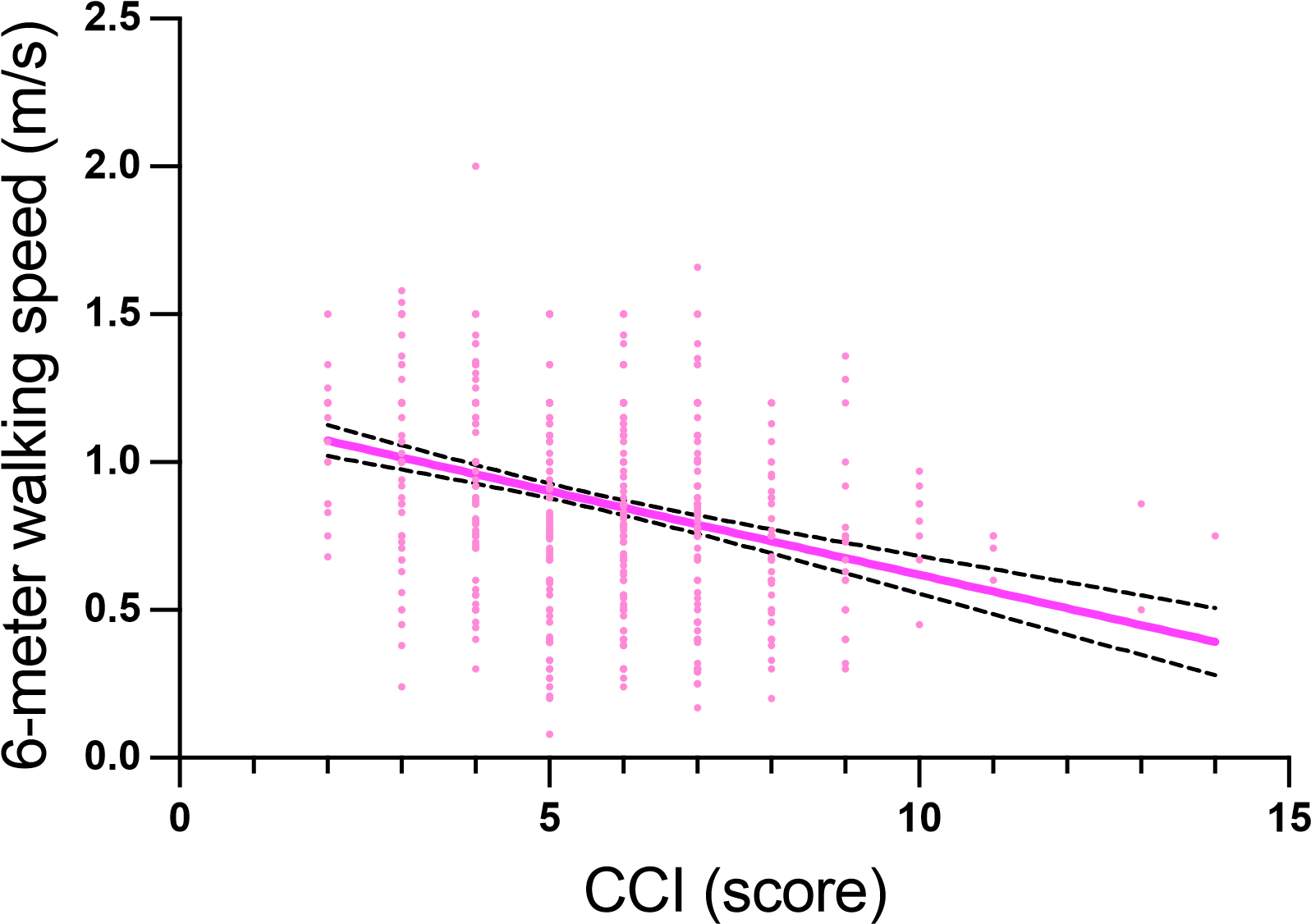

Supplement: Supplementary file 3 — Supplementary Material 3 [file 12891_2024_7412_MOESM3_ESM.docx]
