# Supplementary material for: Correlational analysis of sarcopenia and multimorbidity among older inpatients
Source: BMC Musculoskelet Disord. 2024 Apr 22;25:309. doi: 10.1186/s12891-024-07412-2 (PMC11034126; doi:10.1186/s12891-024-07412-2)
Supplement: Supplementary file 4 — Supplementary Material 4 [file 12891_2024_7412_MOESM4_ESM.docx]

Additional file 4 The value of the Charlson comorbidity index in predicting sarcopenia and diagnostic parameters

|  | AUC | optimal cutoff value | sensitivity | specificity | *p* |
| --- | --- | --- | --- | --- | --- |
|  | 0.691 | 4.5 | 0.831 | 0.458 | 0.000 |
| Grip strength | 0.708 | 4.5 | 0.812 | 0.498 | 0.000 |
| 6–meter walking speed | 0.695 | 4.5 | 0.816 | 0.467 | 0.000 |
| Sarcopenia | 0.777 | 4.5 | 0.927 | 0.487 | 0.000 |
